# Effect of different doses of esketamine compared with fentanyl combined with propofol on hypotension in patients undergoing painless abortion surgery: a prospective, randomized, double-blind controlled clinical trial

**DOI:** 10.1186/s12871-022-01848-6

**Published:** 2022-09-28

**Authors:** Jiejuan Chen, Xiaohua Zou, Bailong Hu, Yang Yang, Feng Wang, Qian Zhou, Minhuan Shen

**Affiliations:** 1 School of Anesthesiology, Guizhou Medical University, Guiyang City, Guizhou Province China; 2 Department of Anesthesiology, Affiliated Hospital of Guizhou Medical University, Guiyang City, Guizhou Province China

**Keywords:** Esketamine, Opioid, Propofol, Painless abortion, Analgesia, Hypotension

## Abstract

**Background:**

Opioids analgesics commonly used in abortion procedures are associated with respiratory and circulatory depression. Esketamine is a N-methyl-D-aspartate receptor (NMDA) antagonist and a common analgesic. The drug has several advantages including rapid onset and offset and it causes minimal cardiorespiratory depression. However, studies have not explored the effects of esketamine in patients undergoing painless abortion surgery. Therefore, the present study sought to evaluate the effect of different doses of esketamine compared with the effect of fentanyl on incidence of perioperative hypotension in patients undergoing painless abortion surgery and to explore the optimal esketamine dose for this population.

**Methods:**

A total of 178 female patients undergoing painless abortion surgery were enrolled to the current study. The patients were aged 18–45 years, had a body mass index (BMI) of 18–28 kg m^− 2^ and a class I or II physical status as determined using the American Society of Anesthesiologists (ASA) system. Patients were randomly assigned to four groups as follows: group F (*n* = 45) in which patients underwent intravenous (IV) administration of 1 μg kg^− 1^ fentanyl followed by IV administration of 2 mg kg^− 1^ propofol, and group EL, group EM and group EH (*n* = 45, 44, 44) with patients receiving IV administration of 0.2 mg kg^− 1^, 0.25 mg kg^− 1^, 0.3 mg kg^− 1^ esketamine, respectively, followed by IV administration of 2 mg kg^− 1^ propofol. The primary outcome of the study was the incidence of hypotension whereas secondary outcomes included incidence of adverse events, perioperative changes of vital signs, anesthesia induction time, recovery time and dischargeable time, propofol addition, as well as patient, surgeon and anesthesiologist satisfaction levels.

**Results:**

The findings showed that the incidence of hypotension was significantly lower in subjects in group EL, group EM and group EH (0, 0, 0%) relative to the incidence in patients in group F (20%) (χ^2^ = 19.648; *P* = 0.000). In this study, the incidence of hypoxia of subjects in group EL, group EM and group EH (0, 2.3, 2.3%) was significantly lower compared with that of patients in group F (11.1%) (χ^2^ = 8.622; *P* = 0.035). The findings indicated that the incidence of somatic motor reactions was significantly lower in participants in group EM and group EH (9.1, 4.5%) relative to that of patients in group F and group EL (26.7, 15.6%) (χ^2^ = 10.254; *P* = 0.016). The results showed that the incidence of nausea and vomiting and potential psychiatric symptoms were significantly higher in patients in group EH (15.9, 11.4%) compared with that of participants in group F (2.2, 0%), group EL (4.4, 0%) and group EM (2.3, 2.3%) (χ^2^ = 7.493; *P* = 0.038 and χ^2^ = 8.248; *P* = 0.003). In this study, the mean arterial pressure (MAP) and heart rate (HR) of subjects in group EL, group EM and group EH were more stable compared with that of patients in group F. Frequency of the additional propofol dose was markedly less in group EM and EH (26.7%, 17,8%) compared with that in group F and EL (9.1, 4.5%) (χ^2^ = 10.254; *P* = 0.016). The findings indicated that the dischargeable time was significantly shorter for patients in group EM compared with that of subjects in group F, group EL and group EH.

**Conclusions:**

The findings of the present study showed that single-dose esketamine (0.25 mg kg^− 1^) effectively decreased incidence of hypotension and total adverse events and reduced the frequency of additional propofol dose required for patients undergoing painless abortion with preservation of physician-patient satisfaction.

## Introduction

The law in China does not restrict abortion. Previous findings indicate that most women with unwanted pregnancy across the world choose abortion for pregnancy termination [1]. Over the past 10 years, the proportion of abortions occurring at under 13 weeks’ gestation, as well as those occurring under 9 weeks’ gestation, and the proportion that are medically induced have all increased [2]. However, traditional abortion methods are associated with discomfort and pain. In severe cases, abortion can lead to loss of the patient’s life [3]. Painless abortion approach significantly reduces the incidence of post-operative adverse reactions, relieves the discomfort caused by the procedure, and reduces the patient’s fear and nervousness compared with traditional abortion procedure performed without anesthetic drugs [4].

Painless abortion is currently widely used globally. Several clinical studies have been conducted to explore methods of improving patient comfort and satisfaction rates as well as evaluate effective anesthesia methods with faster awakening rate, less adverse effects, and high safety. Propofol is an anesthetic with desirable properties such as rapid onset and short duration of action. In addition, propofol is highly lipophilic, has the ability to effectively cross the blood-brain barrier, and is rapidly metabolized and its metabolites effectively excreted through the kidneys [5]. However, propofol has various side effects including pain during injection, addiction, respiratory and circulatory depression [6]. Combination of analgesic drugs with propofol can minimize the dosage and alleviate the adverse effects associated with it [7]. Fentanyl, sufentanil and remifentanil are commonly used analgesic drugs in clinical practice. Opioids effectively suppress the painful stimulation associated with abortion surgery. However, these drugs have side effects such as respiratory depression, decrease in blood pressure, nausea and pain allergy, as well as apnea in severe cases [8–10].

Esketamine is a potent, multimodal dissociative anesthetic. This drug is a N-methyl-D-aspartate receptor (NMDA) antagonist [11]. Esketamine has similar pharmacological characteristics to ketamine. The two drugs exhibit general anesthetic and analgesic effects through inhibition of NMDA receptor activity. In addition, esketamine has mild respiratory depression effect, mild circulatory excitation, and has effects on the diastole of bronchial smooth muscle similar to ketamine. However, esketamine has a higher affinity for NMDA receptors compared with that of ketamine [12]. Moreover, smaller doses of esketamine have more potent anesthetic and analgesic effects relative to ketamine. Previous findings showed that esketamine was twice as potent as ketamine and four times more potent than levocetamine, with mild adverse effects [11]. A randomized parallel-controlled clinical study was conducted to explore the pharmacokinetics and adverse effects of esketamine and ketamine in painless colonoscopy and the findings indicated that the onset of action time of the two drugs was not significantly different. However, the half-life and clearance time of esketamine was significantly shorter than that of ketamine. Furthermore, the findings showed that the pharmacokinetic differences between the two drugs were not significant. The incidence of side effects associated with esketamine was significantly lower than the side effects caused by ketamine. In addition, the degree of side effects caused by esketamine was milder compared with ketamine. The side effects caused by eskatamine mainly included dizziness, agitation, nausea and vomiting, and hypertension. The time taken by patients to awaken and the duration required to regain orientation were significantly shorter for esketamine than for ketamine. Furthermore, the findings showed that a single dose of intravenously administered esketamine (0.5 mg/kg) for approximately 10 s in patients undergoing painless gastroscopy was safe and tolerable. This implies that this drug is more effective and has less side effects in patient undergoing short anesthesia with a rapid turnaround [13]. Several clinical trials report that esketamine has desirable characteristics such as rapid onset and offset, rapid awakening, efficacy with a single injection, low incidence of respiratory depression, high analgesic-hypnotic effect, and rapid antidepressant effect [14–16]. However, studies have not explored efficacy of esketamine in patients undergoing painless abortion surgery. Therefore, the aim of the present study was to evaluate the effect of different doses of esketamine combined with propofol compared with a combination of fentanyl and propofol on hypotension in patients undergoing painless abortion surgery and to explore the optimal esketamine dose for this population.

The hypothesis of the study was that a combination of esketamine and propofol can decrease the incidence of hypotension and adverse events, as well as reduce the propofol dose in patients undergoing painless abortion compared with use of a regimen comprising fentanyl and propofol. In the study, it was postulated that the optimal dose of esketamine for use in painless abortion is 0.25 mg/kg.

## Methods

The current study was a prospective, randomized, double-blind controlled clinical trial. The study was performed following the tenets of the Declaration of Helsinki. Approval to conduct the study was obtained from the institution review board (IRB) of the Affiliated Hospital of Guizhou Medical University (Ref. No. 2022017 K/IRB). Written informed consent was acquired from all patients. The study protocol was registered at chiCTR.org. (ChiCTR2200058324; principal investigator: CHEN Jiejuan; date of registration: April 06/04/2022, no protocol amendment or study changes after start of the trial). This study was conducted according to the relevant CONSORT guidelines.

A total of 178 female pregnant patients scheduled for painless abortion surgery for pregnancy termination were enrolled in the present study. The patients were aged 18–45 years, had a body mass index (BMI) < 28 kg m^− 2^, had grade I-II physical status based on the American Society of Anesthesiologists (ASA) criteria, and were diagnosed with early intrauterine pregnancy. The exclusion criteria for the study were as follows: hearing impairment, previous vaginal delivery, modified equine score grade III and above, recent myocardial infarction (within the previous 7 days), known allergy to fentanyl, propofol or esketamine, severe respiratory diseases (pneumonia or congestive heart failure), hepatic and renal failure, history of neurological disorders (seizure at the onset with postictal residual neurological impairments) and convulsions.

The study was conducted using the emergency equipment such as ventilators, monitoring with defibrillator with external pacing facility, and syringe pumps in the abortion operating room of Affiliated Hospital of Guizhou Medical University. Patients were randomly assigned to four groups using computer-generated random numbers placed into separate opaque envelopes, which were opened by the study investigator just before performing the procedure. The study groups were as follows: group F; patients received a combination of 1 μg kg^− 1^ fentanyl and propofol, group EL; subjects received 0.2 mg kg^− 1^ esketamine and propofol, group EM; patients were administered with 0.25 mg kg^− 1^ esketamine and propofol and group EH; participants received 0.3 mg kg^− 1^ esketamine and propofol. Gynecologists who performed the abortion surgery, all participants, and researchers involved in data collection were blinded to group allocation till the end of the study. All patients underwent fasting for 8 h before going through the procedures.

### Anesthesia management

Standard monitoring was performed after establishing IV access (noninvasive blood pressure, ECG, SpO_2_). A simple oxygen mask (2–4 L/min) was connected to supply oxygen to the patients. Subsequently, anesthesia induction was started after 3 min of oxygen inhalation.

The patients in group F (***n*** = 45) received intravenous (IV) administration of 1 μg kg^− 1^ fentanyl for approximately 30 s, followed by 2 mg kg^− 1^ propofol (IV) for approximately 30 s, and 0.05 mg kg^− 1^ propofol (IV) when the modified observed assessment of alertness/sedation (MOAA/S) score [17] was ≥4 at the start of the surgery or 2 min after IV administration of propofol injection and the propofol dosage repeated as required.

Patients in the EL group (***n*** = 45) intravenously received esketamine 0.2 mg kg^− 1^ for approximately 30 s, followed by 2 mg kg^− 1^ propofol (IV) for about 30 s, and 0.05 mg/kg propofol (IV) was administered when the MOAA/S score was ≥4 at the start of the surgery or 2 min after IV administration of propofol and the propofol dosage repeated appropriately.

Participants in the EM group (***n*** = 44) were intravenously administered with 0.25 mg kg^− 1^ esketamine for about 30 s, followed by 2 mg kg^− 1^ propofol (IV) for approximately 30 s, and 0.05 mg/kg propofol (IV) when the MOAA/S score was ≥4 at the start of the surgery or 2 min after intravenous injection propofol and the propofol dosage repeated as required.

Patients in the EH group (***n*** = 44) received 0.3 mg kg^− 1^ esketamine (IV) for about 30 s, followed 2 mg kg^− 1^ propofol (IV) for approximately 30 s, and 0.05 mg/kg propofol (IV) when the MOAA/S score was ≥4 at the start of the surgery or 2 min after intravenous administration of propofol and the propofol dosage repeated appropriately.

The anesthesiologist determined the required additional dose of propofol required to achieve a MOAA/S score ≤ 2. The sedation level of the patient was assessed by determining the MOAA/S score (in a scale of 0 to 5). In this scale, a score of 0 denotes no response to painful stimuli; 1 indicates response to painful stimuli only (squeezing at the Trapezius site); 2 represents response to light pushing and vibration; 3 denotes response to loud or repeated name calling; 4 indicates delayed response to name calling with normal tone; 5 represents sensitive response to name calling with normal tone. All patients were spontaneously breathing during the process.

Any adverse effects of the treatments were recorded and treated. The adverse effects observed in this study included; 1- hypotension: defined as systolic arterial pressure < 80 mmHg, or decreased baseline systolic blood pressure > 20%, which was treated with 5 mg ephedrine IV; 2- Bradycardia: defined as decrease in HR < 50/min, and was treated through IV administration of atropine 0.5 mg; 3- Hypoxia: defined as SpO_2_ < 90%, treated by assisted manual ventilation using a face mask; 4- Somatic motor reactions, treated with 0.5 mg kg-1 propofol IV; 5- Nausea/vomiting, treated by IV administration of 0.25 mg palonosetron.

Perioperative changes of vital signs including MAP, HR, SPO_2_, and MOAA/S scores were recorded a minute before administration of the drug (T_0_), immediately at MOAA/S ≤ 2 points (T_1_), immediately after the start of the surgery (T_2_), 1 min after the start of the surgery (T_3_), immediately after the end of the surgery (T_4_), and at the moment when the patient woke up from anesthesia (T_5_).

The total additional propofol dose, surgery time, anesthesia induction time, recovery time (time from last dose to time a MOAA/S score ≥ 4 was achieved), dischargeable time, postoperative pain (VAS score at awakening and VAS score at discharge) and the level of injection pain were recorded.

The discharge criteria evaluation was performed by the investigator 10 min after the end of the surgery and every 5 min thereafter until the subject met the criteria for leaving the hospital accompanied by a family member. Criteria for leaving the hospital were as follows: blood pressure and heart rate fluctuations less than 20% of preoperative basal values and stable for ≥10 min; no or mild pain; no or mild nausea and vomiting; no dizziness under sitting position and at the end of walking; subjects able to walk 2 m or more in a straight line on their own after vital signs were stable and monitoring equipment was removed.

Patients were requested to score their satisfaction level during the procedure to record any painful or other undesirable intra-procedural events after full recovery and when the patients were alert enough to express their attitude regarding the intraprocedural events. The surgeon and anesthesiologist also reported their satisfaction levels on the procedure.

The primary outcome of the present study was the incidence of perioperative hypotension. Secondary outcomes included: incidence of perioperative complications such as bradycardia, hypoxia, somatic motor reactions, and nausea/vomiting. Perioperative changes in vital signs, the total additional propofol dose, surgery time, anesthesia induction time, and recovery time, dischargeable time, postoperative pain (VAS score at awakening and VAS score at discharge) and injection pain were determined.

### Statistical analysis

A review of literature showed that there were no similar previous studies on determination of the sample size at the time of designing the study protocol. Therefore, an external pilot study comprising 10 patients in each group was conducted. The results of the pilot study were not included in the full-scale study. The pilot study showed that the incidence of perioperative complications was 3 (30%) in the F group versus 0 (0%) in the EL group, 0 (0%) in the EM group and 0 (0%) in the EH group. The minimal sample size of patients required to get a statistical power level of 0.90 and alpha level at 0.05 was 36 subjects in each group. The calculated sample size was increased by 20% to 45 participants in each group in case of patient dropout.

Collected data were organized, tabulated, and statistically analyzed using SPSS statistical version 25 software (SPSS Inc., USA).

A F-test was conducted to compare the mean values (age, weight and BMI, the surgery time, the anesthesia induction time, the recovery time, and the dischargeable time) of the four groups and data were presented as mean ± standard deviation (SD). Chi-square test was performed for analysis of independent qualitative data. Fischer’s test was used when chi-square test conditions were not met. ASA, side effects and additional propofol dosage data were presented as numbers and percentages and χ^2^ test was conducted. A two-sided *P*-value < 0.05 was considered statistically significant.

## Results

**Fig. 1:**
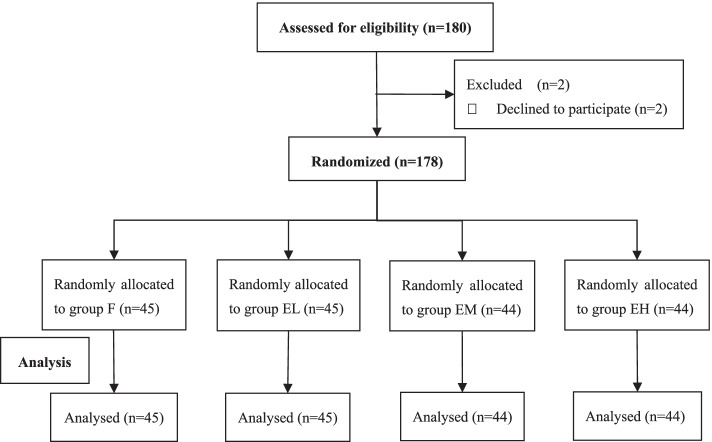
A flow diagram showing the experimental design used in the study

A total of 180 patients were assessed for eligibility in this study based on the inclusion and exclusion criteria. Two patients were excluded and two patients declined to participate in the study. The remaining 178 patients were randomly assigned to the different study groups (Fig. 1).

The demographic characteristics of patients, including age, body weight, BMI, ASA physical status and number of first-time pregnancy abortion were not significantly different among the four groups (Table 1).

**Table 1:** Demographic characteristics of participants

	Group F	Group EL	Group EM	Group EH	F/χ^**2**^	***P***-value ^**a,b**^
**Sample size, n**	45	45	44	44		
**Mean age (±SD) in (years)**	29.8 ± 5.4	30.0 ± 5.7	30.3 ± 6.6	30.0 ± 6.0	0.044	0.988
**Mean weight (±SD) in (kg)**	52.9 ± 6.5	54.1 ± 8.2	54.2 ± 6.7	53.8 ± 5.8	0.329	0.805
**Mean BMI (±SD) in (kg/m**^**2**^**)**	21.2 ± 2.3	21.6 ± 2.8	21.3 ± 2.1	21.2 ± 2.4	0.330	0.804
**ASA, n (%)**						
**I**	33 (73.3%)	33 (73.3%)	34 (77.3%)	32 (72.7%)	0.302	0.960
**II**	12 (26.7%)	12 (26.7%)	10 (22.7%)	12 (27.3%)		
**First-time pregnancy abortion, n (%)**	20 (44.4%)	19 (42.2%)	19 (43.2%)	17 (38.6%)	0.339	0.952

*Abbreviations*: *SD* Standard deviation, *N* number, *Group F* Fentanyl group, *Group EL* Esketamine 0.2 mg/kg group, *Group EM* Esketamine 0.25 mg/kg group, *Group EH* Esketamine 0.3 mg/kg group, *ASA* American Society of Anesthesiologists, *BMI* body mass index

^a^*P*-value for group F compared with group EL, group EM and group EH

^b^F test and Chi-square test was used for data analysis

The incidence of hypotension was significantly lower in group EL, group EM and group EH compared with that of group F (0 (0%), 0 (0%) and 0 (0%) versus 9 (20%)). The results showed that the incidence of hypoxia was significantly lower in groups EL, EM, and EH (0, 2.3, and 2.3%, respectively) compared with that of patients in group F (11.1%). The incidence of somatic motor reactions was significantly lower in groups EM and EH (9.1, 4.5%) relative to that of groups F and EL (26.7, 15.6%). The incidence of nausea and vomiting and potential psychiatric symptoms in group EH (15.9, 11.4%) was significantly higher compared with that of groups F, EL, EM (2.2, 4.4, 2.3% / 0, 0, and 2.3%, respectively) (Table 2).

**Table 2:** Adverse events observed during the procedure

	Group F	Group EL	Group EM	Group EH	χ^**2**^	***P***-value ^**a,b**^
**Sample size, n**	45	45	44	44		
**Hypotension, n (%)**	9 (20%)	0 (0%)^*^	0 (0%)^*^	0 (0%)^*^	19.648	<0.001
**Somatic motor reactions, n (%)**	12 (26.7%)	8 (15.6%)	4 (9.1%)^*^	2 (4.5%)^*#^	10.254	0.016
**Hypoxia, n (%)**	5 (11.1%)	0 (0%)^*^	1 (2.3%)^*^	1 (2.3%)^*^	8.622	0.035
**Nausea and Vomiting, n (%)**	1 (2.2%)	2 (4.4%)	1 (2.3%)	7 (15.9%)^*#$^	7.493	0.038
**Potential psychiatric symptoms, n (%)**	0 (0%)	0 (0%)	1 (2.3%)	5(11.4%)^*#$^	8.248	0.003

*Abbreviations*: *SD* Standard deviation, *N* number, *Group F* Fentanyl group, *Group EL* 0.2 mg/kg group Esketamine, *Group EM* 0.25 mg/kg Esketamine group, *Group EH* 0.3 mg/kg Esketamine group

^a^ *P*-value for group F compared with group EL, group EM and group EH. The symbol ^*^ indicates statistically significant difference compared with group F, ^#^ indicates statistically significant difference compared with group EL, ^$^ indicates that the difference was statistically significant when compared with group EM

^b^ Chi-square test and Fisher’s exact test were used for data analysis

**Fig. 2:**
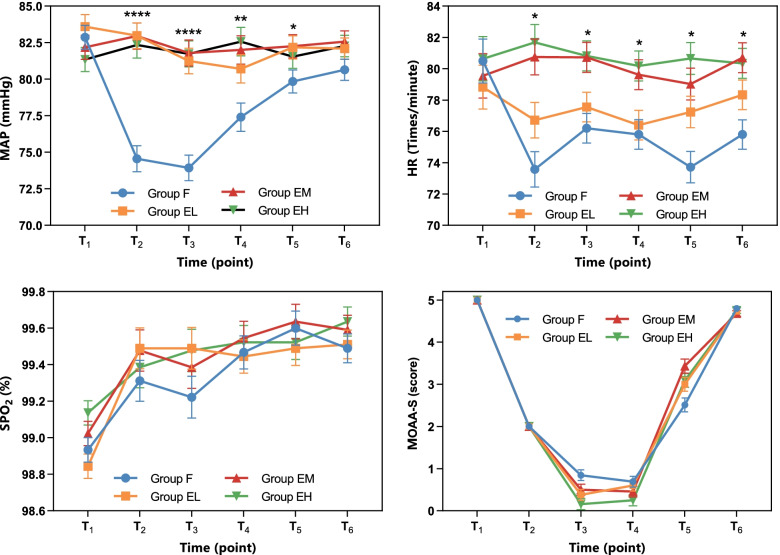
Perioperative changes of vital signs

The magnitude of MAP fluctuations at different T_0_-T_5_ time points was significantly higher for patients in group F relative to that of participants in groups EL, EM and EH (*P* < 0.05). MAP fluctuation levels at T_2_, T_3,_ T_4_ and T_5_ was significantly lower in group F compared with that in groups EL, EM and EH (*P* < 0.05). The results showed that the magnitude of HR fluctuations at different time points of patients in group F was significantly greater relative to that of subjects in groups EL, EM and EH (*P* < 0.05). The HR fluctuation levels at T_2_, T_3_ and T_4_ were significantly lower in group F and EL compared with the HR change in groups EM and EH (*P* < 0.05). In addition, HR fluctuation at T_5_ of subjects in group F and EL was significantly lower relative to that of patients in groups EM and EH (*P* < 0.05). The findings showed that HR fluctuations level at T_5_ of participants in group EL was significantly lower compared with the HR changes of patients in group EH (*P* < 0.05). The HR fluctuation at T_6_ of patients in group F was significantly lower compared with the HR changes of subjects in groups EM and EH (*P* < 0.05). The results showed no significant difference in SPO_2_ and MOAA/S fluctuations among the four groups (Fig. 2).

The recovery time of subjects in group EH was significantly longer compared with that of patients in group F, group EL and EM (11.17 ± 1.64 versus 10.11 ± 1.97, 10.09 ± 2.91 and 9.90 ± 2.25; Table 3). The dischargeable time of patients in group EM was significantly less relative to that of patients in group F, group EL and group EH (11.25 ± 1.31 versus 12.93 ± 1.84, 12.67 ± 2.85, 16.36 ± 2.97, Table 3). The dischargeable time for subjects in group EH was significantly longer compared with that of patients in group F, group EL, and group EM (Table 3). Notably, there was no significant difference in surgery time and anesthesia induction time among the four groups. The frequency of additional propofol doses was significantly lower in subjects in group EM and group EH relative to that of subjects in group F and group EL 4 (9.1%) and 2 (4.5%), versus (12 (26.7%) and 8 (17.8%). The scores on patient satisfaction, surgeon satisfaction and anesthesiologist satisfaction were not significantly different among the four groups (Table 3). The results showed no significant difference in VAS pain scores at awakening and discharge time among the four groups. In addition, the level of injection pain was not significantly different among the four groups.

**Table 3:** Surgery time, anesthesia induction time, recovery time and dischargeable time, frequency of additional propofol dose, patient satisfaction, surgeon satisfaction and anesthesiologist satisfaction in the study

	Group F	Group EL	Group EM	Group EH	F/χ^**2**^	***P***-value ^**a,b**^
**Sample size, n**	45	45	44	44		
**Mean surgery time (±SD) in (min)**	10.33 ± 2.78	10.87 ± 3.20	10.24 ± 2.73	10.64 ± 1.75	0.516	0.672
**Mean anesthesia induction time (±SD) in (min)**	1.55 ± 0.17	1.51 ± 0.10	1.50 ± 0.11	1.50 ± 0.06	2.496	0.062
**Mean recovery time (±SD) in (min)**	10.11 ± 1.97	10.09 ± 2.91	9.90 ± 2.25	11.17 ± 1.64^*#$^	2.897	0.037
**Mean dischargeable time (±SD) in (min)**	12.93 ± 1.84	12.67 ± 2.85	11.25 ± 1.31^*#^	16.36 ± 2.97^*#$^	37.530	0.000
**Frequency of additional propofol, n (%)**	12 (26.7%)	8 (17.8%)	4 (9.1%)^*^	2 (4.5%)^*#^	10.254	0.016
**1**	9 (20.0%)	8 (17.8%)	4 (9.1%)	2 (4.5%)^*#^		
**2**	3 (6.7%)	0(0%)	0 (0%)	0 (0%)		
**Patient satisfaction, n (%)**	39 (86.7%)	42 (93.3%)	42 (95.5%)	36 (81.8%)	5.137	0.162
**Surgeon satisfaction, n (%)**	37 (82.2%)	37 (86.7%)	42 (95.5%)	39 (88.6%)	3.880	0.295
**Anesthesiologist satisfaction, n (%)**	37 (82.2%)	38(84.4%)	42 (95.5%)	36 (81.8%)	4.517	0.209

*Abbreviations*: *SD* Standard deviation, *N* number, *Group F* Fentanyl group, *Group EL* 0.2 mg/kg group Esketamine, *Group EM* 0.25 mg/kg Esketamine group, *Group EH* 0.3 mg/kg Esketamine group

^a^ *P*-value for group F compared with group EL, group EM and group EH. The symbol ^*^ Indicates statistically significant difference compared with group F, ^#^ indicates statistically significant difference compared with group EL, ^$^ implies that the difference was statistically significant when compared with group EM

^b^ F test, Chi-square test and Fisher’s exact test were used for data analysis

## Discussion

In the present prospective, randomized, double-blind controlled clinical trial, participants were assigned to four groups including Fentanyl group, 0.2 mg/kg Esketamine group, 0.25 mg/kg Esketamine group and 0.3 mg/kg Esketamine group. The findings showed that different doses of esketamine combined with propofol significantly reduced the incidence of perioperative hypotension compared with a combination of fentanyl and propofol. The findings of the present study indicated that the patients in the four groups did not present with severe adverse events or adverse reactions that required termination of the trial. In the study, the incidence of hypotension and hypoxia was significantly lower in group EL, group EM and group EH compared with the incidence in group F. Use of opioids and propofol for induction of anesthesia causes hypotension [18–22]. Previous studies report that post-induction hypotension is associated with decreased myocardial contraction and venodilation resulting in reduced venous return. Previous findings indicate that fentanyl anesthesia is associated with arterial dilation with reduced systemic vascular resistance [20–23]. In the present study, patients in the fentanyl group exhibited high intraoperative hemodynamic fluctuations, whereas patients in the esketamine group did not show significant intraoperative changes in blood pressure and heart rate compared with the preoperative values and had insignificant hemodynamic fluctuations. Sigtermans et al. reported that esketamine has sympathomimetic effects [24]. Moreover, a study by Eberl et al. reported that esketamine alleviates hemodynamic fluctuations caused by propofol and maintains hemodynamic smoothness [14]. Opioids cause respiratory depression [25, 26] which can be attributed to the reduction of ventilatory carbon dioxide sensitivity [15]. Moreover, Jonkman et al. reported that esketamine reduces the required opioid dosage as well as alleviates respiratory depression caused by remifentanil and stabilizes breathing in patients [15].

Previous findings indicate that esketamine effectively reduces propofol dosage [14]. The findings of the present study showed that the incidence of somatic motor reactions was significantly lower in patients that received a combination of fentanyl and 0.2 mg kg^− 1^ esketamine relative to the incidence in the groups administered with 0.25 mg kg^− 1^ and 0.3 mg kg^− 1^ esketamine at the same dose of propofol. This implies that an appropriate dose of esketamine significantly reduces propofol dosage. Somatic motor response determines effectiveness of the surgeon in conducting the operation and may lead to an increase in the number of propofol additions during the operation, ultimately reducing the surgeon and anesthesiologist satisfaction rate. The findings showed that the incidence of nausea and vomiting and common psychiatric symptoms was significantly higher in the group that received 0.3 mg kg^− 1^ esketamine compared with that of the groups that received fentanyl, and 0.2 mg kg^− 1^ and 0.25 mg kg^− 1^ esketamine. The psychiatric symptoms observed in the present study mainly included abnormal euphoria and postoperative polyglotism and are attributed to use of excessive dose of esketamine. Nausea, vomiting and psychiatric symptoms affect patient comfort during surgery resulting in reduced patient, surgeon and anesthesiologist satisfaction scores. Notably, the incidence of adverse reactions was low in patients that received 0.2 mg kg^− 1^ and 0.25 mg kg^− 1^ esketamine relative with the other groups. The number of somatic motor reactions and additional propofol doses was significantly lower in patients administered with 0.25 mg kg^− 1^ esketamine compared with the incidence in patients that received 0.2 mg kg^− 1^ esketamine. This finding indicates that administration of esketamine at an appropriate dose reduces the number of adverse reactions and is suitable for outpatient procedures such as painless abortions. However, use of esketamine in patients requires strict respiratory monitoring and anesthetic management.

Esketamine has desirable pharmacological properties such as rapid onset of action, rapid metabolism and rapid awakening [14, 15]. The results in the current study did not show statistically significant difference in anesthesia induction time among the four groups of subjects. Notably, the anesthesia induction duration for the participants of the present study was short. The dischargeable time of patients who received 0.25 mg kg^− 1^ esketamine was significantly shorter relative to that of patients who received 0.1 μg kg^− 1^ fentanyl compared with 0.2 and 0.3 mg kg^− 1^ esketamine.

Injection pain is the most common side effect associated with propofol administration. Propofol-induced injection pain decreases patient comfort and satisfaction. Combination of drugs such as fentanyl and lidocaine with propofol [27], or slowing the push rate effectively reduces propofol-induced injection pain. To the best of our knowledge, no studies have explored the role of esketamine in alleviating propofol-induced injection pain. The present findings did not show significant difference in injection pain and VAS pain scores at postoperative awakening and postoperative discharge times among the four groups, indicating that esketamine reduced propofol-induced injection pain. This finding implies that the analgesic effect of esketamine in painless abortion is similar to that of fentanyl, but has less side effects. Previous findings indicate that esketamine is effective in treatment of acute (perioperative) pain, chronic neuropathic pain and depression [12, 14, 28]. These findings provide a basis for further studies on the clinical application of esketamine during anesthesia induction.

## Limitations of the study

The present study had some limitations. Esketamine and fentanyl are different types of drugs with different mechanisms of action, which may affect the comparison of the two drugs. In the current study, it was not possible to explore the equivalent dose of the esketamine and fentanyl drugs. Therefore, the dosage of the drugs was only determined by pretesting. Moreover, the study population was specific with unique characteristics. All participants were pregnant patients with previous non-vaginal deliveries, which may limit generalization of the study findings to other patient groups. Furthermore, the anesthesia status of the patients was only assessed by the MOAA/S score. Therefore, additional methods such as BIS should be used to verify the findings. In addition, there was no available data for comparison with the present findings as this was the first clinical trial to explore the effect of esketamine in combination with propofol in patients undergoing painless abortion. Despite these limitations, the findings of the study indicate that esketamine use is safe and appropriate dose of esketamine results in favorable outcomes compared with use of fentanyl.

## Conclusions

The findings of the current study showed that a single-dose of esketamine (0.25 mg kg^− 1^) effectively decreased the incidence of hypoxia and total adverse events and reduced the frequency of additional propofol dose required for in patients undergoing painless abortion with no significant effects on physician-patient satisfaction.

## Data Availability

The datasets used and analyzed during the current study are available from the corresponding author on reasonable request.

## Abbreviations

ASA: The American Society of Anesthesiologists
BMI: Body mass index
ECG: Electrocardiogram
EH: Esketamine 0.3 mg kg^− 1^
EL: Esketamine 0.2 mg kg^− 1^
EM: Esketamine 0.25 mg kg-1
F: Fentanyl
HR: Heart rate
IRB: Institution review board
IV: Intravenous injection
MAP: Mean Arterial Pressure
MOAA/S: The modified observe assessment of alertness/sedation
NMDA: N-methyl-D-aspartate receptor
SD: Standard deviation
SPO_2_: oxygen saturation
VAS: Visual Analogue Scale
